# Mercury in honey from the Marche region (central Italy). Risk assessment from human consumption and its use as bioindicator of environmental pollution

**DOI:** 10.1016/j.heliyon.2023.e20502

**Published:** 2023-09-28

**Authors:** Federico Girolametti, Silvia Illuminati, Anna Annibaldi, Behixhe Ajdini, Matteo Fanelli, Cristina Truzzi

**Affiliations:** Department of Life and Environmental Sciences, Università Politecnica delle Marche, Via Brecce Bianche, 60131 Ancona, Italy

**Keywords:** honey, mercury, Italy, Food safety, Risk assessment

## Abstract

Honey is a natural product made by honeybees (*Apis mellifera*) from nectar or honeydew. It is a very popular and appreciated product all over the world as it represents a rapidly available energy source and exerts several beneficial properties for humans. However, it has been demonstrated that honey can be contaminated by potentially toxic elements (PTEs) of natural or anthropogenic origin. Among them, mercury (Hg) represents one of the most dangerous for its toxicity and its capacity to biomagnify along the trophic web. In the present study, 100 honey samples from the Marche Region (Central Italy) produced in the year 2021, were analyzed by thermal decomposition amalgamation atomic absorption spectrometry to determine the Hg content. The overall mean concentration was 0.2 ± 0.2 μg kg^−1^. The results showed that no statistically significant differences were found in Hg content among honey from different pollen origin, but honeydew had a significantly higher Hg content with respect to all other honey samples (0.6 ± 0.3 μg kg^−1^). The Hg content in honey depends mainly on local pollution, while geographical origin did not play a key role. Furthermore, considering the regulatory limits and provisional tolerable weekly intake (PTWIs) identified by FAO/WHO, the Hg Hazard Quotient (HQ) measurement revealed that this product is safe for human consumption.

## 1 Introduction

Honey is a product of bees of the genus *Apis*, which collect nectar from plants or from the secretions of aphids (plant–sucking insects belonging to the genus *Rhynchota*). Honey is a beneficial food to humans, and it is highly appreciated by consumers thanks to its extremely sweet taste and high nutritional value [1]. Since ancient times, honey has been used in food as a sweetener, as a condiment and as a preservative [2] and, due to its chemical characteristics, it has also been used for medical purposes [3]. Consumption of this product is linked to a number of beneficial effects on humans, such as antiviral, antioxidant, anticancer activity and prebiotic properties [4]. Moreover, it exerts immunomodulatory activity in wound healing, has positive effects on metabolism and cardiovascular system and control of human pathogens [[5], [6], [7], [8], [9]].

According to the Food and Agriculture Organization of the United Nations [10] the production of honey globally increased from 778,135 tonnes in 1972 to 1.77 million tonnes in 2021 growing at an average annual rate of 1.78%. China (472,700), Turkey (96,344) and Iran (77,152) are the three main producers [11] and the largest consumers of honey are the Central African Republic, New Zealand and Slovenia (9.62, 5.55 and 4.4 daily grams per capita) [10].

The type of honey may vary according to botanical origin. Monofloral honey is produced by bees that primarily collect nectar from one type of flower. It has distinct organoleptic qualities, such as strongly distinguishing aromas, which are likely derived from nectar [12]. On the other hand, multifloral honey is produced when bees gather nectar from several kinds of flowers. Additionally, there is a variety of honey made from sugar exudates, also known as honeydew or forest honey, which is typically gathered from the sweet exudates of insects.

Mercury (Hg) is a highly toxic metal and can come into contact with humans by ingestion, skin absorption or air inhalation and cause both chronic and acute poisoning [13]. The most toxic forms of mercury are its organic compounds, such as dimethylmercury and methylmercury. Even at low concentrations, exposure to these substances has harmful effects on the blood–brain barrier, the central nervous system, the cellular level, protein synthesis, enzyme activity, and neurophysiological function [[14], [15], [16]].

It is well known that pollutants in the land, water, and air can contaminate bee products [17,18]. Therefore, within approximately 7 km^2^ of its collecting region, honey might be a helpful environmental quality indicator [[19], [20], [21]].

The presence of mercury in honey at the European level is regulated by Commission Regulation (EU) 2018/73 as regards maximum residue levels for mercury compounds in or on certain products and the maximum residue level (MRL) is set at 0.01 mg kg^−1^ [22]. Furthermore, the Joint FAO/WHO Expert Committee on Food Additives (JECFA) set the provisional tolerable weekly intake (PTWI) for inorganic mercury at 4 μg kg^−1^ body weight^−1^ (b.w.) [23].

Although studies have already been carried out on the chemical characterization and accumulation of toxic substances in honey [[24], [25], [26], [27], [28]], few studies have been conducted on mercury content in Italian honey, which is the only country in the world that produces more than 30 varieties of honey [10], besides all of high physicochemical quality [26,[29], [30], [31]]. To the best of our knowledge, only three studies were carried out in Italy on Hg content in honey [[32], [33], [34]].

This study explores three key hypotheses concerning the mercury content in honey samples from the Marche region. The first hypothesis aims to determine the mercury content in honey samples of diverse botanical and geographic origins across the Marche region. By investigating the geographical variability, we aim to identify potential variations in Hg levels based on different floral and environmental factors. The second hypothesis focuses on evaluating the potential risk to human health associated with the consumption of honey containing mercury. We emphasize the novelty of our work by providing updated data and insights on mercury levels in honey from the Marche region, contributing to the current understanding of the health implications for consumers. The third hypothesis explores the application of honey as a bioindicator of environmental pollution, particularly in relation to anthropogenic impacts. By employing an approach that considers the number of inhabitants per municipality and atmospheric PM10 concentration as supporting data, we aim to explore the suitability of honey as a sensitive indicator of environmental pollution, with a particular focus on human-induced influences. Additionally, the findings may offer valuable insights for producers, helping them make informed decisions about honey production practices and potential environmental factors that could impact their products.

## 2 Materials and method

### 2.1 Sampling activity

**Fig. 1:**
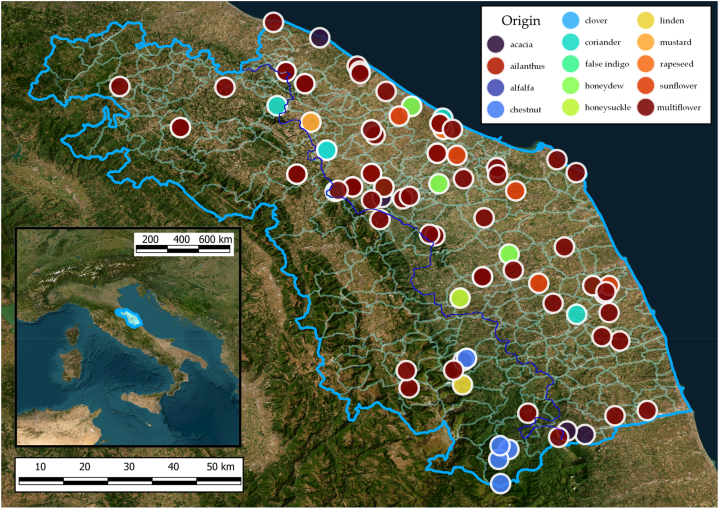
Type of honey collected from the Marche Region (Central Italy). Blue line shows the boundary between the mid hilly coastal belt on the right and high hilly submountain belt on the left. Image concessed by Taffetani F. (SAPROV, Dipartimento di Scienze Agricole, Alimentari e Ambientali, Università Politecnica delle Marche, Ancona, Italy).

The honey samples were delivered by the producers to the Agenzia Servizi Settore Agroalimentare delle Marche (A.S.S.A.M., Ancona, Italy) in the framework of the “Marche Honey Quality Award” event in 2021. Of these, a total of 100 samples (44 monofloral and 56 multiflower honey) have been collected for the determination of Hg. Monofloral honey came from acacia (*Robinia pseudoacacia*, n = 8), ailanthus (*Ailanthus altissima*, n = 1), alfalfa (*Medicago−Sativa*, n = 1), chestnut (*Castanea sativa*, n = 5), clover (*Trifolium* spp., n = 1), coriander (*Coriandrum sativum*, n = 7), false indigo (*Amorpha fruticosa*, n = 1), honeysuckle (*Sulla coronaria*, n = 2), linden (*Tilia* spp., n = 1), mustard (*Sinapis Arvense*, n = 1), rapeseed (*Brassica napus*, n = 2) and sunflower (*Helianthus annuus*, n = 9). Also, five honeydew samples were included. A melissopalynological study of the honey samples was performed in order to guarantee the labeled botanical origin [35]. Aliquots of 50 g were sampled in PE decontaminated vessels [36], shipped to the laboratory and stored at −20 °C protected from light. The geographical sampling region was divided into two belts based on the floristic and phytogeographical characterization of the Marche region [37]: the mid hilly coastal belt (*n* = 74) and high hilly submountain belt (*n* = 26) (Fig. 1).

### 2.2 Laboratory and apparatus

All analytical procedures were carried out in a laboratory with an ISO 5 clean room and laminar flow. A specific cleaning technique using an HCl (34−37% superpure, Carlo Erba, Milano, Italy) 1:10 (v/v) solution was utilized to first decontaminate all scalpels, microspoons, and spatulas that came into touch with the samples [36]. A microanalytical balance (XS205 Mettler Toledo, Greifensee, Switzerland, readability, 0.01 mg; repeatability standard deviation, 0.015 mg) was used to weigh the samples. Decontaminated PE spoons were used as tools to prepare the samples and the variable volume micropipettes with neutral tips were from Brand (Transferpette, Wertheim, Germany). A Milli−Q water system (Merck Millipore, Darmstadt, Germany) was used to obtain ultrapure water. The accuracy of the analytical methodology applied was tested by means of the certified reference material DORM−2 (Dogfish Muscle Reference Material, NRCC, Ottawa, ON, Canada).

### 2.3 Mercury determination

The Hg content in honey samples was determined with thermal decomposition amalgamation atomic absorption spectrometry (TDA AAS), without pre-treatment, using a direct mercury analyzer (DMA−1, FKV, Milestone, Sorisole, Italy). The sample was defrosted at room temperature and homogenized with a Teflon spatula for 5 min, ensuring that as little air as possible was stirred into the honey [38]. About 0.2 g of samples were accurately weighed into a quartz tube and directly placed into the instrument autoinjector. An ultrapure air stream (99.998% purity) transports the sample to the catalytic tube, where it is dried at 250 °C for 60 s, and decomposed at 650 °C for 120 s. The combustion byproducts were taken out and the Hg vapors were captured in a gold amalgamator. The Hg concentration was determined by measuring the absorbance at 253.7 nm after Hg desorption at 650 °C for 60 s and passage in a flow spectrophotometric cell [[39], [40], [41], [42]]. The cell with 0.03–200 ng of Hg detection range (5−200 μg kg^−1^, linearity: R^2^ = 1) was employed. The estimated limits of detection (LOD) and quantitation (LOQ) were 0.03 and 0.11 μg kg^−1^, respectively. The calibration curve method was used to determine the Hg content [43]. Since the concentration was in some cases lower than the LOD, in order to enhance instrumental sensitivity to the detected concentrations, an enrichment method was used to concentrate mercury from several aliquots (n = 3) in the amalgamator and release it at the end of the runs. DORM−2 certified reference material was used to evaluate the analytical quality of measurements. The mean experimental Hg value of 4.54 ± 0.06 mg kg^−1^ dry weight (dw) was consistent with the certified reference material's mean value of 4.64 ± 0.26 mg kg^−1^ dw (p > 0.05) (Δ (%) = −2.3%), indicating a good analytical accuracy. Replicate analysis of the same samples were used to conduct repeatability (<5%). By analyzing the same material over six days reproducibility was examined <10%. The Hg concentration of a blank was subtracted from the measured concentration in samples in order to account for potential Hg contamination during the study. All analyses were carried out in triplicate.

### 2.4 Statistical analysis and visualization

The results are expressed as μg kg^−1^ fresh weight, mean ± standard deviation (min–max). It is well known that the estimation of standard deviation, and so the precision of the method, when only few samples are available, is qualitatively unreliable. So, to measure the analytical error in types of honey where only two samples were available, the pooled standard deviation% (RSDp%) was used instead of the normal standard deviation [30]. Statistical analyses were performed using the RStudio software (R version 4.2.2) and the “ggplot2” package. Each sample group (divided based on type, origin or geographical area) was compared using a one−way analysis of variance (ANOVA): honey type with less than three samples were excluded from this statistical analysis. Multiple comparisons post-test was made using Tukey's test at 95% confidence level. In order to test the homogeneity of the variance, Levene's test was applied. In case of statistically significant differences in the variance, the Welch correction was applied to the ANOVA test. Geographic maps with georeferenced data were produced using GIS software (QGIS version 3.28).

### 2.5 Health hazard estimation

The honey safety was evaluated by comparing the concentrations obtained with the legal values that set limits for this contaminant in the honey matrix. Additionally, the potential non−carcinogenic health risk associated with element exposure through consumption was estimated based on the hazard quotient (HQ) measured by comparing the average daily intake dose (ADD) to the corresponding daily intake reference dose (RfD) (Equation (1) and (2)) [44]:

(1) $$\text{HQ}=\frac{\text{ADD}}{\text{RfD}}$$

(2) $$\text{ADD}=\frac{C\times \text{IR}}{\text{BW}}$$

where C (mg kg^−1^) is the mean concentration of the Hg in honey, IR is the average honey consumption (2.7 g person^−1^ day^−1^) [45], BW is the average body weight of an adult (70 kg), and RfD (mg kg^−1^ day^−1^) is the daily intake reference dose suggested by World Health Organization (Joint FAO/WHO Expert Committee on Food Additives) [23]. An HQ of less than 1 shows no significant risk of non−carcinogenic effects on consumers.

## 3 Results

**Fig. 2:**
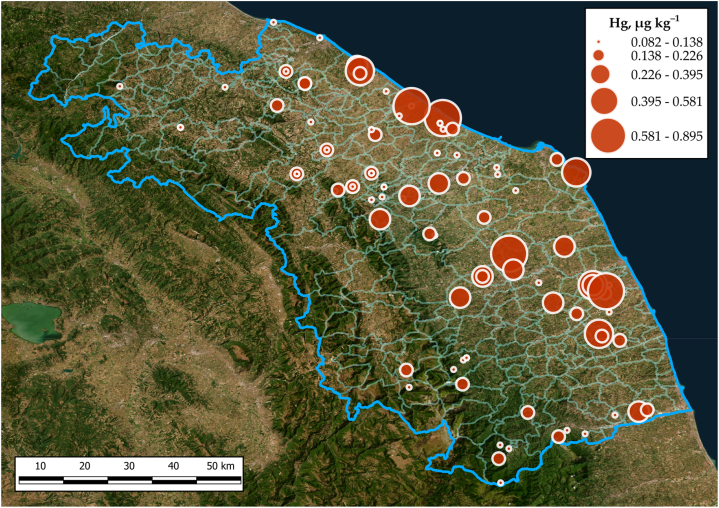
Hg content (μg kg^−1^) in honey collected from the Marche Region (Central Italy).

The comprehensive dataset for samples with honey type, geographical area of origin, province of origin, municipality and Hg content (μg kg^−1^) is shown in Table 1. 20% of the samples reported a concentration below the LOQ. Hg level in different honeys in the Marche Region is graphically reported in Fig. 2. The overall mean Hg content was 0.2 ± 0.2 μg kg^−1^. The highest value (0.9 ± 0.03 μg kg^−1^) was recorded in the honeydew from the municipality of Villa Potenza (Macerata Province), while the lowest value (0.082 ± 0.001 μg kg^−1^) was registered in sunflower honey, from the municipality of Sant’Elpidio a Mare (Fermo province), located both in the central–southern part of the region.

**Table 1:** Hg content in honey expressed as μg kg^−1^ in relation to type, geographical area, province and municipality of origin with number of inhabitants.

Botanical origin	Geographic Area	Province	Municipality (n. inhabitants)^a^	Hg, μg kg^−1^
Acacia	High hilly submountain belt	Ascoli Piceno	Cavignano (76)	0.102 ± 0.006
	High hilly submountain belt	Ascoli Piceno	Piagge (1029)	0.121 ± 0.002
	High hilly submountain belt	Fermo	Sarnano (3042)	0.13 ± 0.05
	High hilly submountain belt	Macerata	Sarnano (3042)	0.13 ± 0.01
	Mid hilly coastal belt	Ancona	Rosora (1818)	0.106 ± 0.005
	Mid hilly coastal belt	Ancona	Senigallia (44019)	0.123 ± 0.005
	Mid hilly coastal belt	Fermo	Sant'Elpidio a Mare (16503)	0.21 ± 0.01
	Mid hilly coastal belt	Pesaro Urbino	Monte San Bartolo (10000)	0.09 ± 0.01
Ailanthus	Mid hilly coastal belt	Fermo	Ponzano di Fermo (1632)	0.54 ± 0.02
Alfalfa	Mid hilly coastal belt	Ancona	Madonna del Piano (188)	0.1 ± 0.01
Chestnut	High hilly submountain belt	Ascoli Piceno	Acquasanta Terme (2494)	0.113 ± 0.003
	High hilly submountain belt	Ascoli Piceno	Quintodecimo (120)	0.15 ± 0.02
	High hilly submountain belt	Ascoli Piceno	Tallacano (9)	0.13 ± 0.01
	High hilly submountain belt	Ascoli Piceno	Venamartello (9)	0.12 ± 0.006
	High hilly submountain belt	Macerata	Sarnano (3042)	0.138 ± 0.007
Clover	Mid hilly coastal belt	Pesaro Urbino	Montalfoglio (17)	0.165 ± 0.007
Coriander	High hilly submountain belt	Ancona	Arcevia (4231)	0.116 ± 0.003
	High hilly submountain belt	Pesaro Urbino	Isola del Piano (543)	0.168 ± 0.002
	Mid hilly coastal belt	Ancona	Madonna del Piano (188)	0.107 ± 0.002
	Mid hilly coastal belt	Ancona	Senigallia (44019)	0.73 ± 0.03
	Mid hilly coastal belt	Ancona	Serra de'Conti (3559)	0.144 ± 0.002
	Mid hilly coastal belt	Fermo	Rapagnano (1908)	0.142 ± 0.005
	Mid hilly coastal belt	Pesaro Urbino	Montalfoglio (17)	0.13 ± 0.01
False indigo	Mid hilly coastal belt	Ascoli Piceno	Spinetoli (7213)	0.39 ± 0.03
Honeydew	High hilly submountain belt	Pesaro Urbino	Pergola (5784)	0.213 ± 0.003
	Mid hilly coastal belt	Ancona	Jesi (39137)	0.379 ± 0.004
	Mid hilly coastal belt	Ancona	Senigallia (44019)	0.72 ± 0.01
	Mid hilly coastal belt	Fermo	Montegranaro (12523)	0.581 ± 0.007
	Mid hilly coastal belt	Macerata	Villa Potenza (2086)	0.9 ± 0.03
Honeysuckle	Mid hilly coastal belt	Ancona	Poggio San Marcello (685)	0.113 ± 0.007
	Mid hilly coastal belt	Macerata	Tolentino (17872)	0.3 ± 0.2
Linden	High hilly submountain belt	Ascoli Piceno	Campolungo (153)	0.14 ± 0.04
Mustard	Mid hilly coastal belt	Pesaro Urbino	Sant'Ippolito (1475)	0.112 ± 0.003
Sunflower	Mid hilly coastal belt	Ancona	Francavilla Trecastelli (2002)	0.12 ± 0.01
	Mid hilly coastal belt	Ancona	Monte San Vito (6684)	0.113 ± 0.006
	Mid hilly coastal belt	Ancona	Morro d'Alba (1798)	0.083 ± 0.004
	Mid hilly coastal belt	Ancona	Osimo (34687)	0.123 ± 0.008
	Mid hilly coastal belt	Ancona	Sant'Angelo di Senigallia (945)	0.12 ± 0.03
	Mid hilly coastal belt	Ancona	Serra de'Conti (3559)	0.086 ± 0.001
	Mid hilly coastal belt	Ancona	Serra de'Conti (3559)	0.11 ± 0.01
	Mid hilly coastal belt	Fermo	Sant'Elpidio a Mare (16503)	0.082 ± 0.001
	Mid hilly coastal belt	Macerata	Corridonia (1467)	0.12 ± 0.01
Rapeseed	Mid hilly coastal belt	Ancona	Sant'Angelo di Senigallia (945)	0.087 ± 0.009
	Mid hilly coastal belt	Ancona	Senigallia (44019)	0.107 ± 0.001
Multiflower	High hilly submountain belt	Ancona	Arcevia (4231)	0.165 ± 0.003
	High hilly submountain belt	Ancona	Rotorscio (200)	0.4 ± 0.2
	High hilly submountain belt	Ascoli Piceno	Castel Trosino (20)	0.15 ± 0.02
	High hilly submountain belt	Ascoli Piceno	Roccafluvione (1850)	0.15 ± 0.02
	High hilly submountain belt	Macerata	Piobbico (2020)	0.11 ± 0.01
	High hilly submountain belt	Macerata	Valfornace (909)	0.152 ± 0.009
	High hilly submountain belt	Macerata	Vallestretta di Ussita (383)	0.088 ± 0.004
	High hilly submountain belt	Pesaro Urbino	Carpegna (1642)	0.11 ± 0.02
	High hilly submountain belt	Pesaro Urbino	Pergola (5784)	0.121 ± 0.009
	High hilly submountain belt	Pesaro Urbino	Pergola (5784)	0.126 ± 0.009
	High hilly submountain belt	Pesaro Urbino	Schieti (396)	0.126 ± 0.006
	High hilly submountain belt	Pesaro Urbino	Urbania (6836)	0.127 ± 0.004
	High hilly submountain belt	Pesaro Urbino	Urbania (6836)	0.125 ± 0.009
	Mid hilly coastal belt	Ancona	Agugliano (4645)	0.094 ± 0.006
	Mid hilly coastal belt	Ancona	Agugliano (4645)	0.126 ± 0.009
	Mid hilly coastal belt	Ancona	Castelbellino (4919)	0.27 ± 0.01
	Mid hilly coastal belt	Ancona	Colle Aprico (17)	0.113 ± 0.006
	Mid hilly coastal belt	Ancona	Corinaldo (4767)	0.181 ± 0.006
	Mid hilly coastal belt	Ancona	Filottrano (8917)	0.177 ± 0.007
	Mid hilly coastal belt	Ancona	Jesi (39137)	0.158 ± 0.008
	Mid hilly coastal belt	Ancona	Madonna del Piano (188)	0.12 ± 0.01
	Mid hilly coastal belt	Ancona	Maiolati Spontini (5986)	0.097 ± 0.004
	Mid hilly coastal belt	Ancona	Mergo (996)	0.121 ± 0.008
	Mid hilly coastal belt	Ancona	Montacuto (267)	0.143 ± 0.008
	Mid hilly coastal belt	Ancona	Monte Conero (4000)	0.6 ± 0.3
	Mid hilly coastal belt	Ancona	Morro d'Alba (1798)	0.092 ± 0.007
	Mid hilly coastal belt	Ancona	Poggio San Marcello (685)	0.108 ± 0.004
	Mid hilly coastal belt	Ancona	Sant'Angelo di Senigallia (945)	0.11 ± 0.005
	Mid hilly coastal belt	Ancona	Senigallia (44019)	0.205 ± 0.002
	Mid hilly coastal belt	Ancona	Senigallia (44019)	0.2 ± 0.02
	Mid hilly coastal belt	Ancona	Senigallia (44019)	0.153 ± 0.003
	Mid hilly coastal belt	Ancona	Serra de'Conti (3559)	0.135 ± 0.006
	Mid hilly coastal belt	Ascoli Piceno	Castel di Lama (8395)	0.12 ± 0.02
	Mid hilly coastal belt	Ascoli Piceno	Monsampolo del Tronto (4418)	0.15 ± 0.02
	Mid hilly coastal belt	Fermo	Francavilla d'Ete (926)	0.25 ± 0.01
	Mid hilly coastal belt	Fermo	Montegranaro (12523)	0.27 ± 0.01
	Mid hilly coastal belt	Fermo	Monterubbiano (1996)	0.15 ± 0.01
	Mid hilly coastal belt	Fermo	Ponzano di Fermo (1632)	0.18 ± 0.01
	Mid hilly coastal belt	Fermo	Sant'Elpidio a Mare (16503)	0.8 ± 0.02
	Mid hilly coastal belt	Fermo	Servigliano (2171)	0.125 ± 0.009
	Mid hilly coastal belt	Macerata	Cingoli (9584)	0.108 ± 0.003
	Mid hilly coastal belt	Macerata	Cingoli (9584)	0.143 ± 0.001
	Mid hilly coastal belt	Macerata	Macerata (40496)	0.33 ± 0.01
	Mid hilly coastal belt	Macerata	Montelupone (3359)	0.321 ± 0.005
	Mid hilly coastal belt	Macerata	Pollenza (6324)	0.34 ± 0.02
	Mid hilly coastal belt	Macerata	Pollenza (6324)	0.14 ± 0.01
	Mid hilly coastal belt	Pesaro Urbino	Fano (59785)	0.252 ± 0.009
	Mid hilly coastal belt	Pesaro Urbino	Fano (59785)	0.553 ± 0.006
	Mid hilly coastal belt	Pesaro Urbino	Fano (59785)	0.226 ± 0.009
	Mid hilly coastal belt	Pesaro Urbino	Gabicce Mare (5496)	0.108 ± 0.002
	Mid hilly coastal belt	Pesaro Urbino	Mombaroccio (2097)	0.164 ± 0.002
	Mid hilly coastal belt	Pesaro Urbino	Mombaroccio (2097)	0.14 ± 0.02
	Mid hilly coastal belt	Pesaro Urbino	San Costanzo (4545)	0.133 ± 0.005
	Mid hilly coastal belt	Pesaro Urbino	Vallefoglia (14935)	0.107 ± 0.007
	Mid hilly coastal belt	Pesaro Urbino	Vallefoglia (14935)	0.17 ± 0.02
	Mid hilly coastal belt	Pesaro Urbino	Vallefoglia (14935)	0.12 ± 0.04

a n. Inhabitants from official website of individual municipalities.

### 3.1 Mercury content *vs* honey botanical origin

**Fig. 3:**
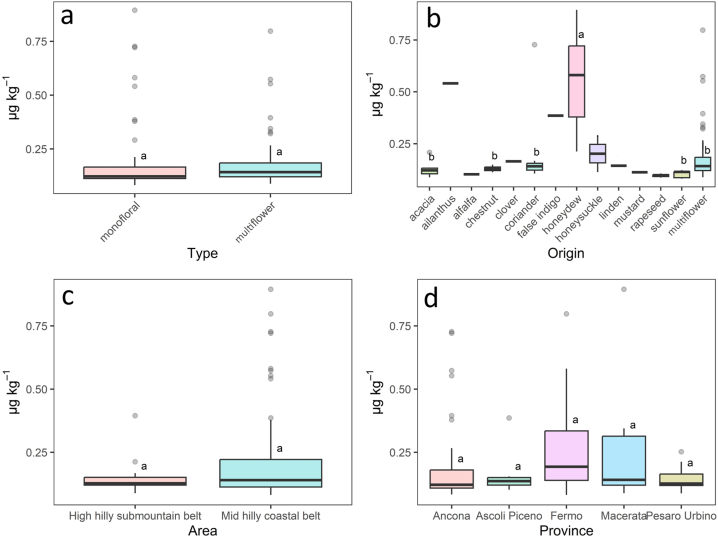
Hg content (μg kg^−1^) in honey collected from the Marche Region (Central Italy) in relation to type (**a**), botanical origin (**b**), geographical area (**c**) and province of origin (**d**). Different letters indicate statistically significant differences among samples groups (P < 0.05). In (**b**), honey types with less than 3 samples were excluded from the statistical analysis.

Hg content in honey collected from the Marche Region in relation to botanical origin is shown in Fig. 3. Regarding the honey type, the mean Hg content of the overall monofloral samples (0.2 ± 0.2 μg kg^−1^) did not show a statistically significant difference (p > 0.05) with respect to the multiflower honey (0.2 ± 0.1 μg kg^−1^) (Fig. 3a). Considering monofloral honey samples (Fig. 3b), the Hg levels follow the order honeydew (0.6 ± 0.3 μg kg^−1^) > ailanthus (0.54 ± 0.02 μg kg^−1^) > false indigo (0.39 ± 0.03 μg kg^−1^) > coriander (0.2 ± 0.2 μg kg^−1^) > honeysuckle (0.2 ± 0.2 μg kg^−1^) > clover (0.165 ± 0.007 μg kg^−1^) > linden (0.14 ± 0.04 μg kg^−1^) > chestnut (0.13 ± 0.01 μg kg^−1^) > acacia (0.13 ± 0.04 μg kg^−1^) > mustard (0.112 ± 0.003 μg kg^−1^) > sunflower (0.11 ± 0.02 μg kg^−1^) > alfalfa (0.10 ± 0.01 μg kg^−1^) > rapeseed (0.100 ± 0.007 μg kg^−1^). Honeydew showed the highest mercury content, statistically different from honey acacia (p = 0.0001), chestnut (p = 0.0004), coriander (p = 0.005), sunflower (p = 0.0001) and multiflower (p = 0.0001). Statistical differences were tested between six honey types because others had too small sample number (<3).

### 3.2 Mercury content *vs* honey geographical origin

Honey samples for this study were taken from several municipalities located within the Marche region. Considering mercury content in relation to the two different geographical macro areas reported, the high hilly submountain belt and the mid hilly coastal belt (0.14 ± 0.06 μg kg^−1^ and 0.2 ± 0.2 μg kg^−1^, respectively), no statistically significant difference (p > 0.05) was evidenced (Fig. 3c). Provincial differentiation also did not seem to influence the distribution of this contaminant as no statistically significant difference (p > 0.05) was found between Hg concentrations in samples collected in the different provinces. The Hg content was, from north to south: 0.14 ± 0.04, 0.2 ± 0.2, 0.2 ± 0.2, 0.2 ± 0.2 and 0.15 ± 0.08 μg kg^−1^ in Pesaro Urbino, Ancona, Macerata, Fermo and Ascoli Piceno provinces, respectively (Fig. 3d).

### 3.3 Risk assessment

Commission Regulation (EU) 2018/73 regulates the maximum residue level (MRL) of Hg in honey, set at 0.01 mg kg^−1^ [22]. As demonstrated, none of the samples analyzed in this study exceeded this limit (Table 1). The average Hg content (0.2 μg kg^−1^) was around 2% of the MRL.

**Fig. 4:**
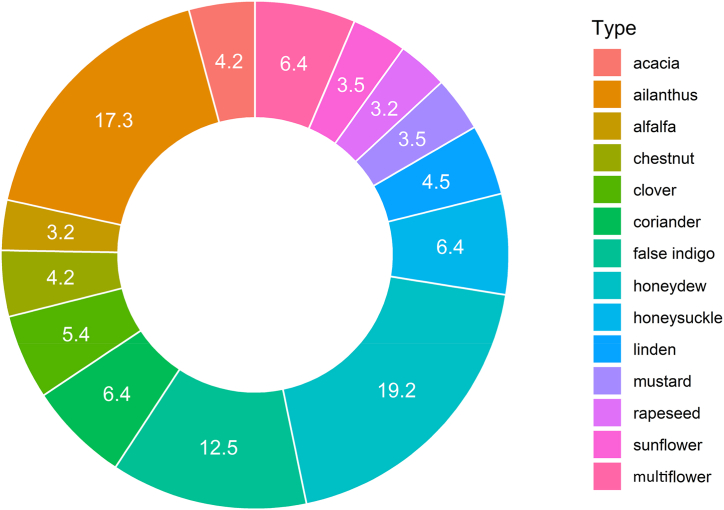
Mercury Hazard Quotient (HQ), expressed as percentage (*vs* the sum of all HQ), in relation to honey botanical origin in the Marche Region (Central Italy).

Hazard Quotient (HQ) was calculated for all honey samples of different botanical origin, and it was always much less than 1. HQ followed the order honeydew (5.8 x 10^−4^) > ilanthus (5.2 x 10^−4^) > false indigo (3.8 x 10^−4^) > coriander (1.9 x 10^−4^) > honeysuckle (1.9 x 10^−4^) > multiflower (1.9 x 10^−4^) > clover (1.6 x 10^−4^) > linden (1.4 x 10^−4^) > chestnut (1.3 x 10^−4^) > acacia (1.2 x 10^−4^) > mustard (1.1 x 10^−4^) > sunflower (1.1 x 10^−4^) > alfalfa (9.7 x 10^−5^) > rapeseed (9.7 x 10^−5^). Fig. 4 showed the HQ percentage (*vs* the sum of all HQ) for each specific honey type.

## 4 Discussion

As components of the daily diet, natural products are now increasingly more and more desired and the idea that they are healthy and safe is becoming increasingly popular among consumers. Honey has a pro–health effect that is used in natural medicine and is also highly appreciated for its taste. It is easily available in grocery stores, herbal stores, pharmacies and directly in apiaries. Despite its beneficial effects, honey is vulnerable to the accumulation of potentially toxic elements (PTEs) and is therefore recognized as an indicator of environmental contamination [17,21,32,46].

### 4.1 Mercury in honey: comparison with literature

Hg concentration in honey from different countries is reported in Table 2. Considering the natural variability of environmental samples, the results of this study are in line with the values found in honey samples from Europe, i.e., Italy, Croatia, Slovakia, Poland, and Turkey.

**Table 2:** Hg content in honey from various countries.

Area	Type	n	Hg (μg kg^−1^) (range)	Reference
Central Italy	All honey	100	0.19 ± 0.16 (0.0815–0.8950)	This study
	Monofloral	44	0.2 ± 0.2 (0.08–0.90)	
	Multiflower	56	0.2 ± 0.1 (0.09–0.80)	
Central Italy	Mainly multiflower	21	7 ± 9 (<1−34)	[32]
Italy	Mono- and multifloral	72	0.19 ± 0.17 (0.04–1.46)	[33]
Poland	All	108	0.43 ± 0.41 (0.01–1.71)	[45]
	Acacia	10	0.41 ± 0.32 (0.01–1.03)	
	Phacelia	4	0.36 ± 0.18 (0.11–0.52)	
	Buckwheat	15	0.28 ± 0.28 (0.07–1.11)	
	Linden	15	0.35 ± 0.28 (0.01–0.97)	
	Dandelion	2	0.12 ± 0.13 (0.03–0.22)	
	Goldenrod	5	0.36 ± 0.54 (0.02–1.30)	
	Rapeseed	10	0.25 ± 0.20 (0.01–0.71)	
	Sunflower	2	0.27 ± 0.24 (0.10–0.44)	
	Honeydew	13	0.72 ± 0.46 (0.07–1.55)	
	Multifloral	30	0.54 ± 0.53 (0.06–1.71)	
	Heather	2	0.29 ± 0.26 (0.11–0.47)	
Poland	All	32	0.37 ± 0.33 (0.02–1.55)	[47]
	Lipowy	5	0.52 ± 0.31 (0.16–0.97)	
	Multifloral	9	0.31 ± 0.21 (0.06–0.70)	
	Acacia	2	0.33 ± 0.04 (0.30–0.36)	
	Goldenrod	3	0.04 ± 0.04 (0.02–0.09)	
	Rape	3	0.37 ± 0.30 (0.18–0.71)	
	Honeydew	3	1.02 ± 0.49 (0.59–1.55)	
	Buckwheat	7	0.19 ± 0.11 (0.07–0.35)	
Croatia	Multiflower	54	2.72 (1–315)	[48]
Slovakia (uncontaminated area)			(50–212)	[49]
Slovakia (contaminated area)			(1–3)	
Aliaga, Turkey		5	n.d.	[50]
Central Cuba	Singing bean	16	<0.010	
	Linen vine	17	<0.010	
	Black mangrove	16	<0.010	
	Christmas vine	18	<0.010	
	Morning glory	16	<0.010	
Zhejiang province, China	All	48	1.65 ± 0.14	[52]
	Acacia	6	2.51 ± 0.19	
	Linden	6	4.00 ± 0.31	
	Citrus	6	0.80 ± 0.07	
	Multifloral	6	2.23 ± 0.21	
	Litchi	6	1.26 ± 0.09	
	Loquat	6	0.55 ± 0.04	
	Jujube	6	0.34 ± 0.02	
	Yellow box	6	1.51 ± 0.12	
South Korea (urban)		1	3249	[57]
South Korea (agricultural)		1	1009	
South Korea (mountain)		1	665	
Singida, Tanzania		90	10.28 (0.38–31.69)	[18]
Nigeria		9	654 ± 10	[53]
Libya		24	(21–100)	[54]
Malesia	Tualang	2	43 ± 47	[55]
	Gelam	2	14 ± 13	
	Pineapple	2	19 ± 20	
	Borneo	2	56 ± 62	
	Kelulut	3	22 ± 16	
	Manuka	3	26 ± 2	
	Commercial Y	3	13 ± 0	
	Commercial Z	3	2 ± 0	

n.d.: not detected.

This study showed that a statistically significantly higher concentration of Hg was recorded in honeydew compared to other types of honey. In Central Italy, a study conducted in the Province of Pesaro Urbino reported significantly higher values than ours (7 ± 9 μg kg^−1^) and found no differences between multiflower honey and acacia honey and honeydew [32]. However, this may be due to the limited number of samples collected for acacia honey (n = 1) and honeydew (n = 1) in that study. Hg content in Italian honey has been investigated also by Quinto et al. (2016) [33], who recorded a mean concentration of 0.19 ± 0.17 μg kg^−1^, confirming our results. In a recent research conducted by Fischer et al. (2022) [45] in honey from Poland, the highest Hg concentration was found in honeydew (0.72 ± 0.46 μg kg^−1^), with a result similar to ours (0.6 ± 0.3 μg kg^−1^), and for honey of other botanical origins the level was always below 0.5 μg kg^−1^, as in this study (except for ailanthus, slightly higher). This trend was observed by another Polish recent study carried out by Brodziak−Dopierała et al. (2021) [47], which discovered the highest content of Hg in honeydew (1.02 ± 0.49 μg kg^−1^). In both studies, the results associated with monofloral honey were wide−ranging depending on botanical origin, whereas reported average concentrations in multifloral honey of 0.54 ± 0.53 [45] and 0.31 ± 0.21 μg kg^−1^ [47], were slightly higher but consistent with our results (0.2 ± 0.1 μg kg^−1^). In honey from Central Europe, a higher concentration of Hg has been found compared to our samples, up to 315 μg kg^−1^ in Croatia [48] and 212 μg kg^−1^ in a contaminated site in Slovakia [49]. In Turkey, Hg in honey has not been detected [50]. In Cuban honey, Hg content were registered with values always lower than 0.01 μg kg^−1^ [51]. Other studies conducted in Asia, such as the one conducted in China by Ru et al. (2013) [52], showed levels of Hg far higher than ours: the average concentration of Hg in acacia honey was 2.51 ± 0.19 μg kg^−1^, in linden honey 4.00 ± 0.31 μg kg^−1^ and in multiflower honey of 2.23 ± 0.21 μg kg^−1^, from 10− to 30−fold higher than honey samples from this study (0.13 ± 0.04, 0.14 ± 0.04 and 0.2 ± 0.1 μg kg^−1^, respectively). The study carried out in South Korea, showed a Hg content of 3−4 orders of magnitude higher with respect to all other studies. Finally, studies conducted in Tanzania [18], Nigeria [53], Libya [54] and Malesia [55], revealed higher average Hg concentrations than the result of the present study or results from European honey. Consequently, it can be deduced that the mercury content in honey is very heterogeneous on a wide geographical scale. One limitation of our study stems from the comparison of honeys of different botanical origins collected from various locations within a wide area, which may introduce variability due to potential differences in environmental factors, floral sources, and bee foraging behaviors. The number of samples (n = 100) is high, but they are collected from 66 different municipalities, so in most cases we have only one or two samples per municipality. This fact, together with the high variability of honey types, did not permit to perform a statistical comparison among honeys of the same botanical origin collected in different areas.

### 4.2 Source of contamination and risk assessment

**Fig. 5:**
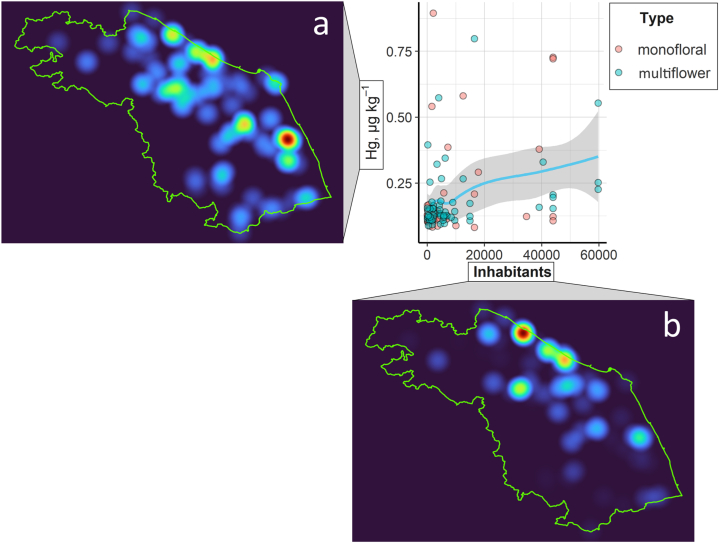
Honey Hg content *vs* number of inhabitants: 7 km radius concentration map of Hg content (μg kg^−1^) in honey samples (**a**) *vs* concentration map of inhabitants located in the municipalities near the honey sampling stations (**b**).

As one of the most widespread and effective pollinators, bees are one of the most crucial components of agriculture. The ability of bees to accumulate high levels of pollutants even in clean regions such as natural reservoirs, even when the hive is located far away from any potential source of contamination is well established [56]. Furthermore, it has been demonstrated in several studies that even plants are affected by environmental pollution and can transfer their contaminant content into hives through pollen collection [46]. Mercury is one of the most harmful among toxic elements, as it can bioaccumulate and biomagnify along with the trophic chain and exerts toxic effects even at very low concentrations. Considering the botanical origin, plant leaves are known to contain higher concentrations of PTEs than flowers and pollen, which would explain why, in our study as well as in the literature, the origin of honeydew makes it the most contaminated by Hg. The Marche Region is located in the central part of Italy and embraces a mountainous area, a hilly area and a coastal area (Adriatic Sea), so the number of inhabitants varies greatly among geographical areas. Since a larger number of people means a greater environmental impact in terms of the number of industries, buildings and vehicular traffic, it is reasonable to think that population density affects the distribution of mercury in honey. In fact, in the study carried out by Meli et al. (2015) [32] in the northern province of the Marche Region (Pesaro Urbino province), higher PTEs concentrations in honey were found in areas with a higher density of inhabitants and greater vehicular traffic. Furthermore, although reporting extremely higher values than this study, an accumulation trend was also identified in South Korea for honey collected from high−populated urban areas rather than agricultural or mountain areas [57]. In the present research, to study the possible correlation between Hg concentration in honey and density of inhabitants, the Hg content of all samples have been mapped as a function of the number of inhabitants per municipality, revealing a statistically significant (p = 0.0009) linear positive correlation (r = 0.33) (Fig. 5). A statistically significant linear positive correlation was found also both for monofloral (r = 0.38, p = 0.01) and multiflower (r = 0.31, p = 0.02) honey. Moreover, to strengthen this outcome, we performed a correlation analysis between honey Hg content and the concentration of atmospheric particulate matter PM10 (μg m^−3^) in the monitoring stations located in the same municipality where the honey samples were taken. These data were obtained from the 2021−year reports of the Regional Agency for the Protection of the Environment (ARPAM) [58]. Unfortunately, only 8 sites from the ARPAM reports coincided with our sampling zones. i.e. Roccafluvione, Macerata, Arcevia, Urbino, Jesi, Fano and two sites in Ancona (Mount Conero and Mantacuto). In any case, an interesting increasing linear trend was found with the equation [Hg] = 0.0162[PM10]+0.0566, (R = 0.48). The results of this work show that there are municipalities where the Hg concentration in honey is higher than others. This data may be an indication of pollution in these areas due to an increased anthropogenic presence that can be considered a possible source of contamination for the honey matrix. Confirming this, the annual bulletins published by the ARPAM recorded higher levels of particulate matter [59] and lower river quality [60] in the proximity of the municipalities with the largest populations. This result fits in with the fact that bees visit flowers in the area a few kilometers away from the hive [[19], [20], [21],^59^]. In the light of this debate, it is clear that the different Hg concentration found in honey samples from Marche region depends not so much on vegetation belts that characterize the territory, as on small−scale contamination effects. Also, Quinto et al. (2016) [33] found that honey from areas with declared high levels of contaminants showed a higher content of PTEs, completely suppressing the effect of the different botanical origin. Therefore, the Hg content in honey can be used to identify the most environmentally impacted areas in the regional territory showing that honey can be used as a bioindicator of local pollution. However, the samples analyzed in this study were completely safe from the point of view of human nutrition as no sample exceeded the legal limit set at 0.01 mg kg^−1^ [22]. Furthermore, the non–carcinogenic risk by HQ calculation from mercury exposure due to the consumption of the honey analyzed showed that there is no risk for consumers. A similar risk analysis was conducted also in the study published by Ru et al. (2013) [52], where the HQ values of individual metals ingested via honey consumption were all lower than 1 (between 10^−2^ and 10^−4^) in the following order: As > Pb > Zn > Hg > Cd > Cu. The authors stated that generally, when HQ values are below 10^−4^ the associated risk can be considered acceptable depending on the exposure situation and circumstances. It is well known that exposure to potentially toxic elements other than Hg (such as e.g. Pb, As, Cd, Cr) can result not only from multiple foods, but also from inhalatory and dermal sources of exposure. In addition, numerous other chemical contaminants of different natures (POPs, pesticides, PCBs, PAHs, emerging contaminants …) can lead to cumulative effects, resulting from the addition or interaction of their individual effects. For this reason, the use of the Hazard Index (HI), calculated as the sum of the individual HQ, must be considered for the overall risk assessment.

## 5 Conclusion

The mercury content in honey from the Marche region (Central Italy) varies greatly depending on the raw material of which the product is made, pollen or honeydew. Whereas no statistically significant differences were found among honey from different pollen origin, honeydew turns out to be the honey with the significantly highest mercury content of six types tested. Furthermore, there is evidence that in municipalities with a greater number of inhabitants, and therefore a stronger anthropic impact, the Hg content in honey tends to be higher with respect to honey collected from less urbanized areas, suggesting that this product can be used as a bioindicator of environmental pollution. We have however to underline that the limitation of our study stems from the comparison of honeys of different botanical origins collected from various locations within a wide area, which may introduce variability due to potential differences in environmental factors, floral sources, and bee foraging behaviors. Finally, all the honey samples analyzed were found to be safe for human consumption both by comparison with the Hg regulatory limit and taking into account the Hg Hazard Quotient (HQ) index. Given the increasing impact of humans on the environment, continuous monitoring of this toxic element in food matrices that are suitable as bioindicator is necessary to maintain a product quality guarantee for the consumer. Our study provides valuable and complementary information to the existing knowledge, contributing to a more comprehensive understanding of mercury levels in honey and their potential implications for human health and environmental pollution in the Marche region.

## Author contribution statement

Federico Girolametti: Conceived and designed the experiments; Performed the experiments; Analyzed and interpreted the data; Wrote the paper.

Silvia Illuminati: Analyzed and interpreted the data.

Anna Annibaldi: Analyzed and interpreted the data; Contributed reagents, materials, analysis tools or data.

Behixhe Ajdini, Matteo Fanelli: Performed the experiments.

Cristina Truzzi: Conceived and designed the experiments; Analyzed and interpreted the data; Contributed reagents, materials, analysis tools or data; Wrote the paper.

## Data availability statement

Data included in article/supp. material/referenced in article.

## Declaration of competing interest

The authors declare that they have no known competing financial interests or personal relationships that could have appeared to influence the work reported in this paper.

## References

[1] Khan F.R., Abadin Z.Ul, Rauf N. Honey (2007). Nutritional and medicinal value. Int. J. Clin. Pract..

[2] Crane E. (1999).

[3] Molan P.C. (1999). Why honey is effective as a medicine. 1. Its use in modern medicine. Bee World.

[4] Viuda-Martos M., Ruiz-Navajas Y., Fernández-López J., Pérez-Álvarez J.A. (2008). Functional properties of honey, propolis, and royal jelly. J. Food Sci..

[5] Simon A., Traynor K., Santos K., Blaser G., Bode U., Molan P. (2009). Medical Honey for Wound Care—Still the ‘Latest Resort’? Evidence-Based Complementary and Alternative Medicine.

[6] Hossen MdS., Ali MdY., Jahurul M.H.A., Abdel-Daim M.M., Gan S.H., Khalil MdI. (2017). Beneficial roles of honey polyphenols against some human degenerative diseases: a review. Pharmacol. Rep..

[7] Miguel M., Antunes M., Faleiro M. (2017). Honey as a complementary medicine. Integr. Med. Insights.

[8] Kamaruzzaman M.A., Chin K.-Y., Mohd Ramli E.S. (2019). A review of potential beneficial effects of honey on bone health. Evidence-Based Complementary and Alternative Medicine 2019.

[9] Nweze A., Olovo V., Innocent Nweze E., Okechukwu John O., Paul C. (2020). Honey Analysis - New Advances and Challenges.

[10] FAO; IZSLT (2019).

[11] (2021). Knoema Honey Production in the World.

[12] Schievano E., Morelato E., Facchin C., Mammi S. (2013). Characterization of markers of botanical origin and other compounds extracted from unifloral honeys. J. Agric. Food Chem..

[13] Graeme K.A., Pollack C.V. (1998). Heavy metal toxicity, Part I: arsenic and mercury. J. Emerg. Med..

[14] Syversen T., Kaur P. (2012). The toxicology of mercury and its compounds. J. Trace Elem. Med. Biol..

[15] Aaseth J., Wallace D.R., Vejrup K., Alexander J. (2020). Methylmercury and developmental neurotoxicity: a global concern. Curr Opin Toxicol.

[16] Zanoli P., Truzzi C., Veneri C., Braghiroli D., Baraldi M. (1994). Methyl mercury during late gestation affects temporarily the development of cortical muscarinic receptors in rat offspring. Pharmacol. Toxicol..

[17] Bogdanov S. (2006). Contaminants of bee products. Apidologie.

[18] Maggid A.D., Kimanya M.E., Ndakidemi P.A. (2014). The contamination and exposure of mercury in honey from singida, Central Tanzania. Am J Res Commun.

[19] Przybyłowski P., Wilczyńska A. (2001). Honey as an environmental marker. Food Chem..

[20] Almeida-Silva M., Canha N., Galinha C., Dung H.M., Freitas M.C., Sitoe T. (2011). Trace elements in wild and orchard honeys. Appl. Radiat. Isot..

[21] Czipa N., Andrási D., Kovács B. (2015). Determination of essential and toxic elements in Hungarian honeys. Food Chem..

[22] Commission Regulation (Eu) (2018).

[23] (2011). FAO Evaluations of the Joint FAO/WHO Expert Committee on Food Additives (JECFA).

[24] Jones K.C. (1987). Honey as an indicator of heavy metal contamination. Water Air Soil Pollut..

[25] Leita L., Muhlbachova G., Cesco S., Barbattini R., Mondini C. (1996). Investigation of the use of honey bees and honey bee products to assess heavy metals contamination. Environ. Monit. Assess..

[26] Truzzi C., Annibaldi A., Illuminati S., Finale C., Scarponi G. (2014). Determination of proline in honey: comparison between official methods, optimization and validation of the analytical methodology. Food Chem..

[27] Solayman Md, Islam MdA., Paul S., Ali Y., Khalil MdI., Alam N., Gan S.H. (2016). Physicochemical properties, minerals, trace elements, and heavy metals in honey of different origins: a comprehensive review. Compr. Rev. Food Sci. Food Saf..

[28] Zhu L., Wang Z., Wong L., He Y., Zhao Z., Ye Y., Fu P.P., Lin G. (2018). Contamination of hepatotoxic pyrrolizidine alkaloids in retail honey in China. Food Control.

[29] Conti M.E., Stripeikis J., Campanella L., Cucina D., Tudino M.B. (2007). Characterization of Italian honeys (Marche region) on the basis of their mineral content and some typical quality parameters. Chem. Cent. J..

[30] Truzzi C., Annibaldi A., Illuminati S., Finale C., Rossetti M., Scarponi G. (2012). Determination of very low levels of 5-(hydroxymethyl)-2-furaldehyde (HMF) in natural honey: comparison between the HPLC technique and the spectrophotometric white method. J. Food Sci..

[31] Truzzi C., Illuminati S., Annibaldi A., Finale C., Rossetti M., Scarponi G. (2014). Physicochemical properties of honey from Marche, Central Italy: classification of unifloral and multifloral honeys by multivariate analysis. Nat. Prod. Commun..

[32] Meli M.A., Desideri D., Roselli C., Benedetti C., Feduzi L. (2015). Essential and toxic elements in honeys from a region of Central Italy. J. Toxicol. Environ. Health.

[33] Quinto M., Miedico O., Spadaccino G., Paglia G., Mangiacotti M., Li D., Centonze D., Chiaravalle A.E. (2016). Characterization, chemometric evaluation, and human health-related aspects of essential and toxic elements in Italian honey samples by inductively coupled plasma mass spectrometry. Environ. Sci. Pollut. Control Ser..

[34] Astolfi M.L., Conti M.E., Ristorini M., Frezzini M.A., Papi M., Massimi L., Canepari S. (2021). An analytical method for the biomonitoring of mercury in bees and beehive products by cold vapor atomic fluorescence spectrometry. Molecules.

[35] Azeredo L. da C., Azeredo M.A.A., de Souza S.R., Dutra V.M.L. (2003). Protein contents and physicochemical properties in honey samples of Apis mellifera of different floral origins. Food Chem..

[36] Illuminati S., Truzzi C., Annibaldi A., Migliarini B., Carnevali O., Scarponi G. (2010). Cadmium bioaccumulation and metallothionein induction in the liver of the antarctic teleost *Trematomus bernacchii* during an on-site short-term exposure to the metal via seawater. Toxicol. Environ. Chem..

[37] Taffetani F. (2015). Caratterizzazione Floristica e Fitogeografica Del Miele Millefiori Delle Marche. Seminario “Mappatura Delle Aree Nettarifere.” In Proceedings of the C.R.A. Consiglio per la Ricerca e la Sperimentazione in Agricoltura, Istituto Sperimentale per la Zoologia Agraria; Firenze.

[38] Harmonised Bogdanov S. (1997). Methods of the European honey commission. Apidologie.

[39] Droghini E., Annibaldi A., Prezioso E., Tramontana M., Frapiccini E., De Marco R., Illuminati S., Truzzi C., Spagnoli F. (2019). Mercury content in central and southern adriatic Sea sediments in relation to seafloor geochemistry and sedimentology. Molecules.

[40] Truzzi C., Annibaldi A., Girolametti F., Giovannini L., Riolo P., Ruschioni S., Olivotto I., Illuminati S. (2020). A chemically safe way to produce insect biomass for possible application in feed and food production. Int. J. Environ. Res. Publ. Health.

[41] Girolametti F., Frapiccini E., Annibaldi A., Illuminati S., Panfili M., Marini M., Santojanni A., Truzzi C. (2022). Total mercury (THg) content in red mullet (mullus barbatus) from adriatic Sea (central mediterranean Sea): relation to biological parameters, sampling area and human health risk assessment. Appl. Sci..

[42] Girolametti F., Panfili M., Colella S., Frapiccini E., Annibaldi A., Illuminati S., Marini M., Truzzi C. (2022). Mercury levels in Merluccius Merluccius Muscle tissue in the central mediterranean Sea: seasonal variation and human health risk. Mar. Pollut. Bull..

[43] Annibaldi A., Truzzi C., Carnevali O., Pignalosa P., Api M., Scarponi G., Illuminati S. (2019). Determination of Hg in farmed and wild atlantic bluefin tuna (Thunnus thynnus L.) Muscle. Molecules.

[44] Girolametti F., Annibaldi A., Illuminati S., Damiani E., Carloni P., Truzzi C. (2023). Essential and potentially toxic elements (PTEs) content in European tea (camellia sinensis) leaves: risk assessment for consumers. Molecules.

[45] Fischer A., Brodziak-Dopierała B., Bem J., Ahnert B. (2022). Analysis of mercury concentration in honey from the point of view of human body exposure. Biol. Trace Elem. Res..

[46] Girotti S., Ghini S., Ferri E., Bolelli L., Colombo R., Serra G., Porrini C., Sangiorgi S. (2020). Bioindicators and biomonitoring: honeybees and hive products as pollution impact assessment tools for the mediterranean area. EuroMediterr J Environ Integr.

[47] Brodziak-Dopierała B., Mendak-Oleś P., Fischer A. (2021). Occurrence of mercury in various types of honey. Environ. Med..

[48] Bilandžić N., Đokić M., Sedak M., Kolanović B.S., Varenina I., Končurat A., Rudan N. (2011). Determination of trace elements in Croatian floral honey originating from different regions. Food Chem..

[49] Toporcák J., Legáth J. (1992). Kul’ková J levels of mercury in samples of bees and honey from areas with and without industrial contamination. Vet. Med..

[50] Matin G., Kargar N., Buyukisik H.B. (2016). Bio-monitoring of cadmium, lead, arsenic and mercury in industrial districts of izmir, Turkey by using honey bees, propolis and pine tree leaves. Ecol. Eng..

[51] Alvarez-Suarez J.M., Giampieri F., Damiani E., Astolfi P., Fattorini D., Regoli F., Quiles J.L., Battino M. (2012). Radical-scavenging activity, protective effect against lipid peroxidation and mineral contents of monofloral Cuban honeys. Plant Foods Hum. Nutr..

[52] Ru Q.-M., Feng Q., He J.-Z. (2013). Risk assessment of heavy metals in honey consumed in zhejiang province, southeastern China. Food Chem. Toxicol..

[53] Idoko J.O., jege K.O., Haruna B.S., Tifwa P.A., Musa W.O. (2018). Evaluation of heavy metals in honey from brinin-gwari (Nigeria). Journal of Chemical Society of Nigeria.

[54] Salama A.S., Etorki A.M., Awad M.H. (2019). Determination of physicochemical properties and toxic heavy metals levels in honey samples from west of Libya. Journal of Advanced Chemical Sciences.

[55] Kek S.P., Chin N.L., Tan S.W., Yusof Y.A., Chua L.S. (2017). Classification of honey from its bee origin via chemical profiles and mineral content. Food Anal. Methods.

[56] Perugini M., Manera M., Grotta L., Abete M.C., Tarasco R., Amorena M. (2011). Heavy metal (Hg, Cr, Cd, and Pb) contamination in urban areas and wildlife reserves: honeybees as bioindicators. Biol. Trace Elem. Res..

[57] Gizaw G., Kim Y., Moon K., Choi J.B., Kim Y.H., Park J.K. (2020). Effect of environmental heavy metals on the expression of detoxification-related genes in honey bee Apis mellifera. Apidologie.

[58] (2023). ARPA Marche *La Qualità Dell’aria Nella Regione Marche*.

[59] (2022). ARPA Marche *L’aria Nelle Marche Nel 2022: I Primi Dati*.

[60] (2023). ARPA Marche *Fiumi*.

